# Cognitive Inflexibility in Obsessive-Compulsive Disorder and Major Depression Is Associated with Distinct Neural Correlates

**DOI:** 10.1371/journal.pone.0059600

**Published:** 2013-04-24

**Authors:** Peter L. Remijnse, Odile A. van den Heuvel, Marjan M. A. Nielen, Chris Vriend, Gert-Jan Hendriks, Witte J G. Hoogendijk, Harry B. M. Uylings, Dick J. Veltman

**Affiliations:** 1 Department of Psychiatry, VU University Medical Center, Amsterdam, The Netherlands; 2 Neuroscience Campus Amsterdam, Amsterdam, The Netherlands; 3 Department of Anatomy and Neuroscience, VU University Medical Center, Amsterdam, The Netherlands; 4 Center for Anxiety Disorders Overwaal, Lent, The Netherlands; 5 Department of Psychiatry, Erasmus MC, University Medical Center Rotterdam, Rotterdam, The Netherlands; 6 Division Cognitive Neuropsychiatry and Clinical Neuroscience, School for Mental Health and Neuroscience, University of Maastricht, Maastricht, The Netherlands; Chiba University Center for Forensic Mental Health, Japan

## Abstract

Obsessive-compulsive disorder (OCD) and major depressive disorder (MDD) are frequently co-morbid, and dysfunctional frontal-striatal circuits have been implicated in both disorders. Neurobiological distinctions between OCD and MDD are insufficiently clear, and comparative neuroimaging studies are extremely scarce. OCD and MDD may be characterized by cognitive rigidity at the phenotype level, and frontal-striatal brain circuits constitute the neural substrate of intact cognitive flexibility. In the present study, 18 non-medicated MDD-free patients with OCD, 19 non-medicated OCD-free patients with MDD, and 29 matched healthy controls underwent functional magnetic resonance imaging during performance of a self-paced letter/digit task switching paradigm. Results showed that both patient groups responded slower relative to controls during repeat events, but only in OCD patients slowing was associated with decreased error rates. During switching, patients with OCD showed increased activation of the putamen, anterior cingulate and insula, whereas MDD patients recruited inferior parietal cortex and precuneus to a lesser extent. Patients with OCD and MDD commonly failed to reveal anterior prefrontal cortex activation during switching. This study shows subtle behavioral abnormalities on a measure of cognitive flexibility in MDD and OCD, associated with differential frontal-striatal brain dysfunction in both disorders. These findings may add to the development of biological markers that more precisely characterize frequently co-morbid neuropsychiatric disorders such as OCD and MDD.

## Introduction

Obsessive-compulsive disorder (OCD) and major depressive disorder (MDD) are frequently co-morbid psychiatric disorders [1] that share several features such as symptomatic overlap [2] and clinical improvement following serotonergic antidepressants [3]. Recent neurobiological models of OCD have emphasized abnormal activity in prefrontal cortex (i.e., orbitofrontal cortex (OFC) and dorsolateral prefrontal cortex (DLPFC)), anterior cingulate cortex (ACC), and subcortical (caudate and putamen) brain regions, as well as in (para)limbic structures such as insula and amygdala [4] [5]. In MDD, neurobiological models have similarly outlined prefrontal cortical, paralimbic and subcortical abnormalities, involved in the pathophysiology of this disorder [6] [7]. Despite these commonalities at a clinical and neurobiological level, OCD and MDD clearly differ with regard to symptom constellations [8] and neuropsychological profiles [9] [10]. Thus, it has been stated that a challenge for modern-day neuropsychiatry research is to find common and distinct neurobiological correlates of depression and OCD, i.e. to identify discriminating endophenotypes such as neuropsychological probes for neuroimaging use [6]. A promising neuropsychological paradigm in this context is cognitive flexibility - defined as the ability to rapidly change response strategies upon altering task-relevant information in the environment [11] - that is likely to be impaired in both OCD [12] and MDD [13]. Several ways of operationalizing cognitive flexibility have been introduced in laboratory settings, e.g. intra/extradimensional set shifting and reversal learning [14]. However, such paradigms conflate switching with contingency learning [14], yet feedback-based learning is a cognitive domain that itself may be abnormal in MDD [15] and OCD [16]. A neuropsychological tool for measuring cognitive flexibility uncontaminated by contingency learning is task switching [17]. At a behavioral level, task switching paradigms are have traditionally been associated with a ‘switch cost’, i.e. increased reaction times (RTs) and error rates upon switch trials relative to repeat trials, reflecting enhanced cognitive demands [18]. Human lesion studies have shown increased switch costs during task switching in patients with (especially left-sided) prefrontal cortical damage compared with controls [19] [20]. Moreover, patients with early-stage Huntington's disease [21] and Parkinson's disease [22] displayed increased switch costs on task switching experiments, suggesting intact basal ganglia function is a prerequisite for adequate task switching performance. Finally, functional neuroimaging studies in healthy volunteers have shown activity during task switching - accompanied by behavioral switch costs - in DLPFC [23] [24], medial PFC [23], inferior parietal cortex [23] [24], ACC [24], anterior PFC [25] and putamen [24]. Indeed, these frontal-striatal, parietal and (para)limbic neural networks that constitute the neural substrate of intact task switching are similar to brain circuits supposedly dysfunctional in both MDD [6] [7] and OCD [4] [5].

To our knowledge, only one neuropsychological task switching study in OCD has been published [26] that failed to find increased switch costs in patients with OCD compared with healthy controls, despite numerous reports in the literature of deficits in OCD on related measures of cognitive flexibility, i.e. intra-dimensional set-shifting (Veale et al. 1996), extra-dimensional set-shifting [27] [28] (but see [29]) and reversal learning [30] (but see [31]). Possibly, the large amount of medicated and co-morbid depressed OCD patients in the Moritz et al. study [26] may explain the lack of performance differences between patient and control groups. In MDD, no controlled neuropsychological task switching studies have been published so far.

In a previous functional neuroimaging study by our group directly comparing OCD and MDD, we demonstrated decreased activations in DLPFC, anterior PFC, inferior parietal cortex and ACC in both patients groups relative to healthy controls during affective switching in a reversal learning design. Also, anterior insula activity was found to differ between patient groups, suggesting differential emotion-related neural processing in OCD and MDD [32]. However, as noted previously, (affective) switching and learning are confounded measures in a cognitive flexibility paradigm like reversal learning [14]. Therefore, in order to assess the neural correlates of isolated switching behavior in unmedicated patients with OCD and MDD relative to controls, we conducted the present study using a task switching design in a three-group functional magnetic resonance imaging (fMRI) experiment. Based on the above-reviewed literature and our own between-groups findings [32], we hypothesized impaired task performance in OCD and MDD associated with abnormal activations in DLPFC, anterior PFC, ACC and insula. In addition, we expected differential insula activity between patient groups.

## Methods

### Participants

Eighteen patients with OCD (without MDD), 19 patients with MDD (without OCD), and 29 healthy controls participated in this study. Patients were recruited from psychiatric outpatient clinics and by Internet advertisements. Diagnoses and comorbidity were established by experienced clinicians with the Structured Clinical Interview for DSM-IV Axis-I disorders (SCID) [33]. Exclusion criteria were age below 18 or above 65, the presence of alcohol or substance abuse, and major internal or neurological disorders. In the OCD group, 8 patients were diagnosed with ‘pure’ OCD, and the following disorders were comorbid: posttraumatic stress disorder (N = 1), panic disorder (N = 2), generalized anxiety disorder (N = 4), dysthymic disorder (N = 4), social anxiety disorder (N = 4), opioid abuse in sustained full remission (N = 1), and Tourette's syndrome (N = 1). In the MDD group, twelve patients had MDD only, and comorbid disorders included social anxiety disorder (N = 3), generalized anxiety disorder (N = 1), panic disorder without agoraphobia (N = 1), and pain disorder (N = 1). Healthy controls were screened for the absence of current or past psychiatric and neurological diseases, as well as substance abuse.

Patients and control subjects were free from psychotropic medication for at least two weeks, and in case of fluoxetine or antipsychotic medication for at least one month.

To assess symptom characteristics and severity scores, the Yale-Brown Obsessive-Compulsive Scale [34] (Y-BOCS) was administered in OCD patients only, whereas the Padua-Inventory Revised [35](Padua-IR) was used to measure all participants' obsessive-compulsive (OC) characteristics. To rate the presence and severity of depressive symptoms in all three groups, the Beck Depression Inventory [36](BDI), the 21-item Hamilton Depression Rating Scale [37] (HDRS-21) and the 10-item Montgomery-Asberg Depression Rating Scale [38] (MADRS) were used.

### Ethics statement

The study was conducted in accordance with the principles expressed in the Declaration of Helsinki. All participants were considered to be fully capable and able to provide informed consent, as judged by the experienced clinicians who conducted the interviews. All participants gave written informed consent and the study was approved by the ethical review board of the VU University medical center.

### Task switching paradigm

**Figure 1:**
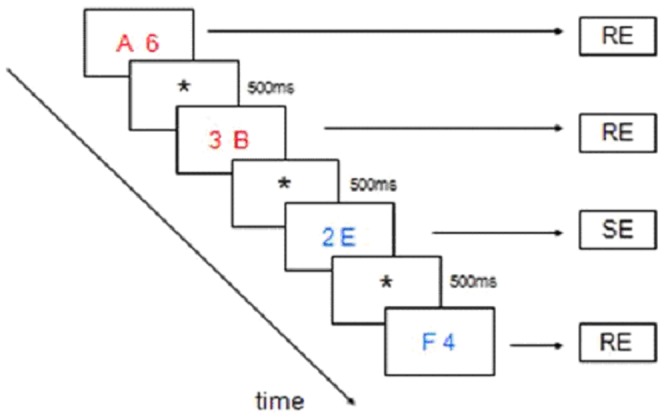
The letter/digit task switching paradigm. In this example (consecutive trials are running from top-left to bottom-right) the events-of-interest are displayed. Subjects are presented two stimuli on each trial, i.e. a letter and a digit, for 4000 ms maximally. Subjects select either stimulus by pressing the left or right button on a button box, after which a fixation cross is presented for 500 ms. Each letter/digit pair is presented in either blue or red color. The trial color cues the task to be performed. In the letter task, subjects indicate whether the letter presented is a vowel or a consonant. In the digit task, subjects indicate whether the digit presented is odd or even. Two consecutive trials never contain the same letter or digit. Trial color changes, and therefore task switching, occurs randomly after 4–6 trials to avoid predictability. The first trials immediately after task switching are defined ‘switch events’ (SEs), all other trials as ‘repeat events’ (REs). Color-task and stimulus-response associations were counterbalanced across participants.

We used a modified self-paced task switching (letter/digit) paradigm based on [40], graphically outlined in figure 1. Each trial consisted of two stimuli - a letter and a digit – presented side by side on a screen, for 4000 ms maximally. Participants selected either stimulus by pressing the left or right button on a button box, after which a fixation cross was presented for 500 ms. Each letter/digit pair was presented in either blue or red color. The trial color cued the task to be performed. In the letter task, participants indicated whether the letter presented was a vowel or a consonant. In the digit task, participants indicated whether the digit presented was odd or even. Letters were taken from the set {a, e, i, u, b, c, d, f} and digits were taken from the set {2, 4, 6, 8, 3, 5, 7, 9}. Two consecutive trials never contained the same letter or digit. Color-task and stimulus-response associations were counterbalanced across participants. Trial color changes, and therefore task switching, occurred randomly after 4–6 trials to avoid predictability. The first trials immediately after task switching were defined as ‘switch events’ (SEs), all other trials as ‘repeat events’ (REs). The task ended after 32 discrimination stages, i.e. after 31 task switches.

Participants received task instructions regarding the color-task and stimulus-response associations, and were encouraged to minimize response RTs and to avoid errors. Participants practiced the task twice, once within at most two weeks before the scanning session using a computer, and the second time in the scanner prior to the actual experiment.

### Imaging procedure

Imaging data were collected using a 1.5-T Sonata MR system (Siemens, Erlangen, Germany) with a standard circularly polarized head coil. Task stimuli were projected on a screen behind the participant's head at the end of the scanner table, visible through a mirror mounted above the subject's head. Two magnet-compatible response boxes were used to record the participant's responses. In order to reduce motion artefacts, the participant's head was immobilized using foam pads.

T2*-weighted echo-planar images (EPI's) with blood oxygenation level-dependent contrast (BOLD) were acquired in each session. Using this sequence with a TR of 2.18 s and a TE of 45 ms, 35 slices (3×3 mm in-plane resolution; 2.5 mm slice thickness; matrix size 64×64) per image were acquired. A whole-brain EPI-image for each participant was also acquired using the same sequence (40–43 slices per image, 3 images in total) as well as a structural image using a 3D coronal T1-weighted sequence (voxel size 1×1×1.5 mm, 160 sections).

### Data analysis

Demographic and behavioral data were analyzed using SPSS software (version 11.5 for Windows; SPSS Inc, Chicago, Ill). Switch costs (SEs minus REs) in each group were computed using paired samples t-tests for mean RTs and error rates. Furthermore, mean RTs and error rates for SEs and REs as well as switch costs were compared between groups using one-way (simple) ANOVAs with group (MDD vs. OCD vs. controls) as between-subject factor and event type (SEs, REs and switch costs) as within-subject factor. Correlations (Pearson's r) were calculated between performance measures and severity of OC symptoms (Padua-IR and Y-BOCS) as well as depression severity (MADRS, BDI, and HDRS-21) in the OCD and MDD group, respectively. Alpha was set at p<0.05.

Imaging analysis was performed using SPM5 software (Wellcome Trust Centre for Neuroimaging, London, UK). Images were reoriented, slice-timed and realigned to the first volume. The resulting mean image was then co-registered to the whole-brain EPI-volume, and images were normalized to MNI-space as defined by a SPM T2* template and spatially smoothed using a 6 mm Full Width at Half Maximum Gaussian kernel. Statistical analysis was carried out in the context of the general linear model, in which SEs were modeled using a delta function convolved with a canonical hemodynamic response function. Error trials were additionally modeled as a regressor of no interest. Contrast images containing parameter estimates for our comparison of interest, i.e., switch vs. repeat trials, were computed at single-subject level and subsequently entered in second-level one-way ANOVAs. Main effects for task and for group were adjusted for the whole-brain search volume using the false discovery rate (FDR) method implemented in SPM [41], and reported at a significance level of p<.05, unless indicated otherwise. Group x task interaction effects were reported at p<.001 uncorrected, masked inclusively with the orthogonal main effect to restrict the search volume to those voxels showing a main effect of task [42] and to obtain a reasonable balance between Type I and Type II error, similar to our previous study in these groups [32]. Finally, we performed regression analyses (reported at p<.001 uncorrected) between OC (Padua-IR and Y-BOCS) and MDD (BDI, MADRS, HDRS-21) severity scores, and task effects in the OCD and MDD group, respectively.

## Results

### Demographic and clinical data

The three groups were adequately matched for age, handedness and educational level, but not for gender (table 1). A one-way ANOVA revealed main effects for all depression severity measures (BDI, MADRS, HDRS-21), due to MDD patients scoring significantly higher than OCD patients, and the latter group scoring significantly higher than healthy volunteers. On the Padua-IR, a one-way ANOVA showed a main effect due to both patient groups scoring significantly higher than the control group, but no significant difference between patient groups. A subsequent analysis of Padua-IR scores in the MDD group demonstrated that these were mainly related to the rumination (N = 13), precision (N = 1), checking (N = 2), and impulses (N = 1) subdimensions, whereas the OCD group showed mixed symptoms, the highest Padua-IR scores being related to the checking (N = 10), rumination (N = 4), washing (N = 1) and precision (N = 1) subdimensions [43].

**Table 1:** Demographic and clinical data for patients with obsessive-compulsive disorder (OCD), patients with major depressive disorder (MDD), and healthy controls.

	OCD (N = 18)	MDD (N = 19)	Controls (N = 29)	Between-groups comparison
	Mean (SD)	Mean (SD)	Mean (SD)	P-value
Sex (Female/Male)	14/4	7/12	20/9	0.02#
Age (range)	33 (19–54)	35 (21–54)	33 (22–53)	0.77‡
Handedness (R/L)	16/2	15/4	25/4	0.67 #
Education (range 1–10)‡	8.5 (1.2)	8.0 (2.1)	8.6 (1.3)	0.42‡
Total Y-BOCS severity score	22.7 (4.9) (range 11–31)			
Number of OCD patients with prior MDD/mean length in months since remission of MDD	8/36			
Padua-IR, mean (S.D.)	58.2 (25.8) (N = 16)	43.9 (32.8) (N = 17)	10.9 (10.2)	<.001‡ MDD = OCD>CO*
BDI	10.7 (6.0) (N = 15)	24.9 (7.1) (N = 18)	1.8 (2.6)	<.001‡ MDD>OCD>CO*
HDRS-21, mean (S.D.)	10.1 (4.6) (N = 13)	20.1 (4.4)	0.6 (1.4)	<.001‡ MDD>OCD>CO*
MADRS, mean (S.D.)	8.8 (6.7) (N = 16)	29.5 (4.7)	0.8 (1.4)	<.001‡ MDD>OCD>CO*

# chi-square ‡ One-way ANOVA * Tukey and Scheffe post-hoc tests.

### Behavioral data

Table 2 shows behavioral data on the task switching paradigm for the three groups. We found a significant switch cost (SEs versus REs) for mean RTs in each of the three groups (controls: 1474 ms vs. 1025 ms, paired samples t-test: t(28) = −11.1; p<.0001. OCD: 1540 ms vs. 1179 ms, t(17) =  −6.8; p<.0001. MDD: 1664 ms vs. 1155 ms, t(18) =  −11.2; p<.0001). We also found a significant switch cost for mean error rates in the healthy control group (9.4 vs. 5.5, t(28) =  −2.5; p = .03), and in the OCD group (4.1 vs. 2.5, t(17) =  −2.0; p = .05), but not in the MDD group (7.0 vs. 5.6, t(18) =  −1.6; p = .12).

**Table 2:** Behavioral data on the task switching paradigm for patients with obsessive-compulsive disorder (OCD), patients with major depressive disorder (MDD), and healthy controls.

Event type	OCD (N = 18)				MDD (N = 19)				Controls (N = 29)				
	RTs (ms)		Error %		RTs (ms)		Error %		RTs (ms)		Error %		
	Mean	SD	Mean	SD	Mean	SD	Mean	SD	Mean	SD	Mean	SD	
Repeat events (REs)	1179	203	2.5	2.4	1155	300	5.6	7.0	1025	220	5.5	5.1	
												
Switch events (SEs)	1540	247	4.1	4.1	1664	359	7.0	8.9	1474	325	9.4	11.2	
												
Switch cost (SE-RE)	T	p	T	p	T	p	T	p	T	p	T	p	
	−6.8	.0001	−2.0	.05	−11.2	.0001	−1.6	.12	−11.1	.0001	−2.5	.03	

One-way ANOVAs showed no significant differences across groups for switch costs on RTs (F(2,63) = 2.2; p = .11) or error rates (F(2,63) = 1.1; p = .33), and a trend significant performance difference across groups for mean RTs on REs (F(2,63) = 2.8; p = .06). Planned comparisons revealed a significant RT difference between OCD patients and controls on REs (ANOVA: F(1,45) = 5.8; p = .02; d = 0.63), a trend significant RT difference between MDD patients and controls on REs (F(1,44) = 3.0; p = .09; d = 0.49), but not between-patient groups RT difference on REs (F(1,35) = 0.08; p = .77; d = 0.15). Furthermore, one-way ANOVAs showed no significant performance difference across groups for mean RTs on SEs (F(2,63) = 2.0; p = 0.13) nor for error rates on REs (F(2,63) = 2.2; p = 0.11), or SEs (F(2,63) = 1.8; p = 0.17). However, planned comparisons showed that OCD patients had a significantly lower RE error rate than controls (F(1,45) = 5.4; p = .02; d = 0.58). We found a trend significant RT difference on SEs between MDD and controls (F(1,44) = 3.6, p = .06; d = 0.60) and a trend significant error rate difference on SEs between OCD and controls (F(1,45) = 3.5; p = .07; d = 0.42).

Correlations between performance measures and disease severity in the MDD group showed a significant positive correlation between MADRS scores and mean RTs on SEs (r = .45; p = .05). In the OCD group, a significant *negative* correlation was found between Y-BOCS scores and error rates on SEs (r = −.55; p = .02) and a trend significant negative correlation between Y-BOCS scores and error rates on REs (r = −.45; p = .06). No significant correlations were found for total Padua-IR scores and performance measures in OCD.

### Imaging data

#### Task and group main effects

**Figure 2:**
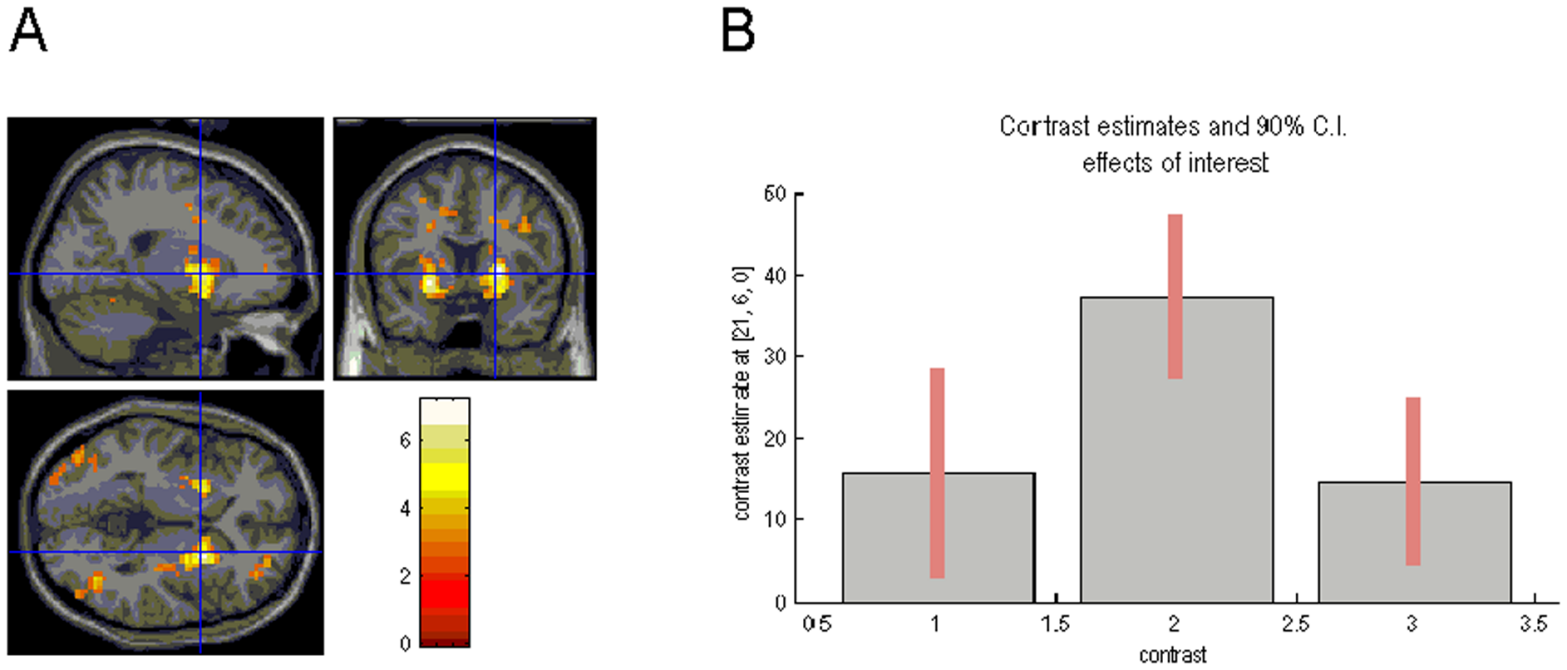
Task main effects and plot of effect sizes. (A) Task main effects for switching, superimposed on sagittal, transaxial and coronal slices from a canonical (MNI [Montreal Neurological Institute] compatible) T1 image as supplied by SPM. Enhanced BOLD responses are shown in the putamen bilaterally. (B) A plot of effect size in the left putamen is displayed for all three groups (MNI coordinates: x = −21, y = 6, z = 0), showing increased activation in this brain area for patients with obsessive-compulsive disorder (OCD) relative to patients with major depressive disorder (MDD) and the control group.

Across groups, main effects of task were found in frontal-striatal circuitry, in particular DLPFC, anterior PFC, and putamen (figure 2A), as well as parietal and occipital brain regions. These activations were also found in the healthy control group, but patient groups failed to show significant BOLD effects in several of these brain regions, i.e. the OCD group lacked activations of anterior PFC and DLPFC, and the MDD group showed no DLPFC and only minimal anterior PFC activations at our a priori threshold (data not shown).

#### Group x task interaction effects

**Figure 3:**
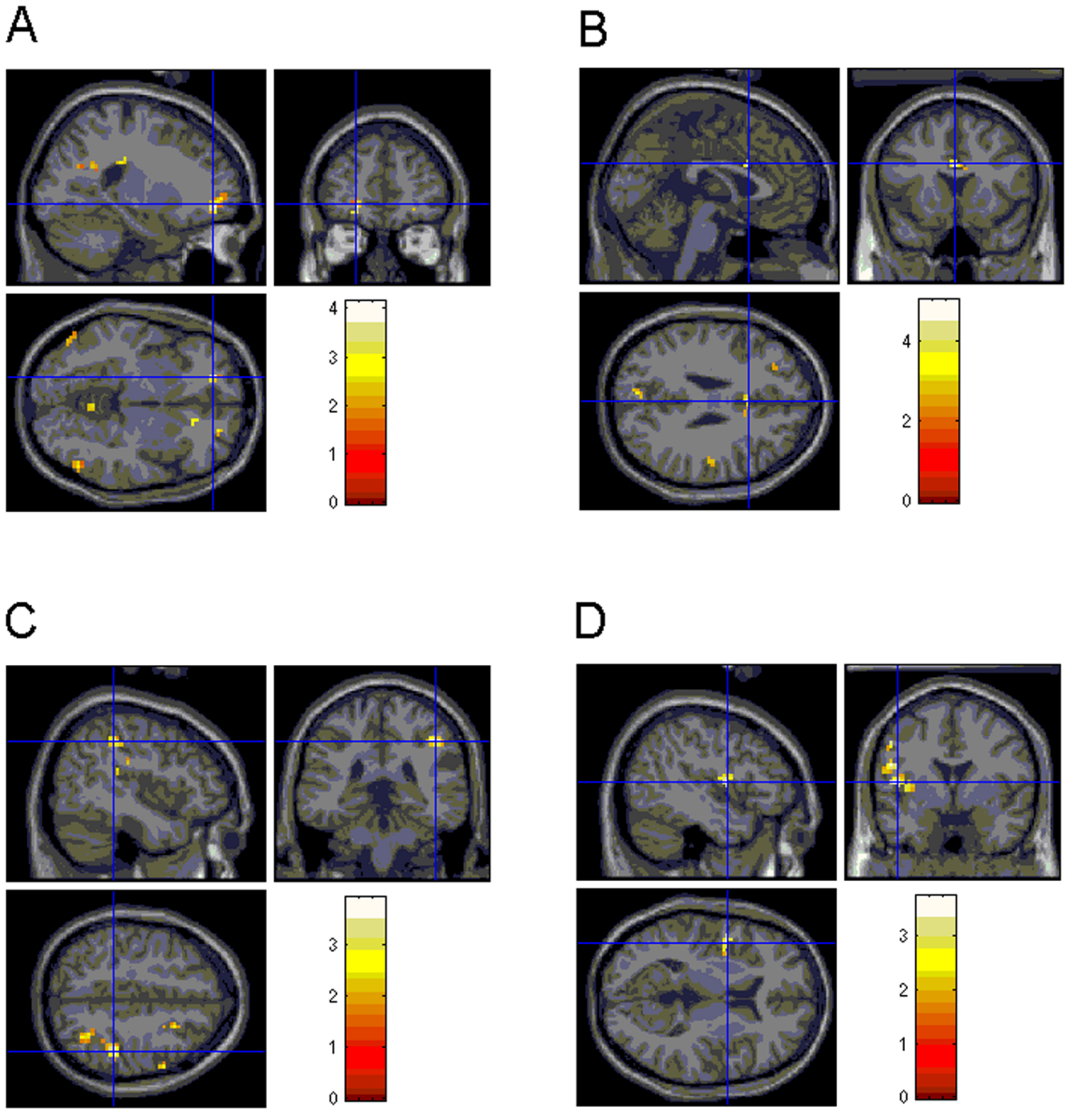
Group by condition (SE vs. RE) differences. (A) enhanced BOLD response in left anterior PFC (controls vs. OCD), (B) in dorsal ACC (OCD vs. controls), (C) in right inferior parietal cortex (controls vs. MDD) and (D) in left insula (OCD vs. MDD).

Healthy control subjects showed increased activity in left anterior PFC (Figure 3A) compared with OCD patients. In contrast, patients with OCD revealed increased BOLD responses in left putamen (figure 2B), bilateral ACC (Figure 3B), and left postcentral gyrus, compared with controls. Similar findings were obtained after omitting four OCD patients with comorbid dysthymia (data not shown). Furthermore, healthy controls demonstrated increased activity in right anterior PFC and right inferior parietal (Figure 3C) hyperactivity relative to MDD patients. No significantly increased activations were found for MDD patients compared with controls. Comparisons between patient groups showed significantly enhanced signal in bilateral putamen, left insula (Figure 3D), left postcentral gyrus, right precuneus, and left supramarginal gyrus for OCD relative to depressed subjects. No significant activation differences were found for MDD versus OCD patients (table 3).

**Table 3:** Group x task interaction effects on the task switching paradigm for the group of patients with obsessive-compulsive disorder (OCD), patients with major depressive disorder (MDD), and healthy controls. All activations at p<.001 uncorrected.

Regions	L/R	Cluster size	MNI coordinates				z-value
			x	y	z		
			**Controls>OCD**				
Anterior PFC	L	2	−27	51	−12		3.50
			**OCD>controls**				
ACC	R	3	0	15	27		4.58
Postcentral gyrus	L	7	−48	−18	30		3.98
Putamen	L	3	−21	6	0		3.33
			**Controls>MDD**				
Parietal inf	R	4	45	−36	45		3.70
Anterior PFC	R	5	21	54	0		3.72
			**MDD>controls No significant activations**				
			**OCD>MDD**				
Postcentral gyrus	L	9	−54	−18		27	4.00
Putamen	L	2	−21	0		6	3.56
	R	3	24	15		3	3.49
Precuneus	R	3	18	−66		48	3.22
Insula	L	2	−36	−3		6	3.43
	L	2	−33	−15		12	3.39
Supramarginal gyrus	L	2	−48	−39		27	3.10
			**MDD>OCD No significant activations**				

#### Regression and covariance analyses

In patients with OCD, we found a significant negative correlation between total Padua-IR scores and left anterior PFC activity (MNI coordinates: −15, 57,3; r = −.56; p = .023), a significant positive correlation between Y-BOCS scores and right ACC activity (6,15,33; r = .56; p = .014), and significant positive correlations between Y-BOCS and Padua-IR scores, and left putamen activity (−21,15,0; r = .70; p = .001; and −27,9,9; r = .67; p = .004; figure 2C and 2D). Patients with MDD showed no significant correlations between depression severity scores and group imaging effects.

Finally, we performed analyses of covariance with gender as a dummy variable to investigate whether the observed group x task interaction effects could be explained by the previously reported skewed male/female ratio across groups. This analysis showed that the described effects persisted after controlling for differences in gender.

## Discussion

The present imaging study is the first to investigate cognitive flexibility uncontaminated by contingency learning in groups of unmedicated patients with OCD and MDD relative to healthy controls. The currently used task switching paradigm yielded a robust switch cost for mean RTs in each group, and for error rates in the OCD and control group. Possibly, we failed to find an error rate switch cost in the MDD group because these patients tended to slow down their responses on SEs, resulting in a near-significant RT difference between MDD and controls on SEs. Contrary to expectations, we failed to find switch cost differences between our patients and healthy controls. This was probably not due to poor performance in our control group, as the mean RT during switch trials in control participants (1.45”) was similar to the Sohn et al. [40] study (1.51”) on which we based our design. However, further between-group performance analyses showed prolonged RTs on REs in the OCD group compared with controls, which was associated with a reduced RE-related error rate in OCD relative to controls. This suggests that slower performance in OCD is compensatory for the sake of accuracy. This OCD-specific behavioral finding is congruent with the (partially trend significant) negative correlations between Y-BOCS scores and error rates on REs and SEs in the OCD group, indicating that switch performance becomes more accurate with increasing OC severity. In contrast, although patients with MDD tended to disproportionally slow down on SEs, RTs and error rates on both REs and SEs in these patients were similar to those in controls. Taken together, the present study provides evidence for differential performance patterns in both patient groups. In OCD, we found OC-severity to be beneficial to accuracy at the expense of prolonged responding during repetition. In contrast, depression severity (as measured by MADRS scores) in MDD was associated with increased response latency during switching without a compensatory effect for accuracy. Our findings of performance differences during repeat rather than switch events may seem surprising, but are in accordance with recent evidence that differential switch costs may result from differences in adaptation during repeat trials [44], cf. [45]. The absence of a switch cost in OCD and MDD patients as observed in the present study is also in agreement with several recent task switching studies in adolescents with OCD [46] [47] or MDD [49]. However, our behavioral findings are at odds with the study of Gu et al. [49] who observed a significantly higher error rate during switch events rather than slowing down during non-switch trials in adult patients with OCD versus healthy controls, which may have been due to differences in task implementation, for example the use of fixed vs. jittered interstimulus intervals. Of note, the current findings of differential accuracy in OCD and MDD patients also extend our previous results using a reversal learning paradigm in these groups [32], in which we failed to observe significant performance differences after excluding OCD participants with comorbid MDD, suggesting that cognitive function alterations rather than impaired motivational processing may distinguish between these disorders.

With regard to imaging results, we found - as hypothesized - abnormal (i.e. attenuated) activity in task-relevant brain areas such as anterior PFC (extending into OFC) during switching both in OCD and MDD, compared with healthy controls. Moreover, in OCD subjects, left-lateralized anterior PFC activity was negatively correlated with total Padua-IR scores. The anterior PFC is a higher-order cognitive brain area and has been implicated in coordinating multiple separate cognitive operations in the pursuit of a higher behavioral goal [50]. Task switching in a letter/digit design may be considered an executive demand integrating several cognitive operations - e.g. reconfiguring a new task set while inhibiting the previous task set, and updating appropriate task-associated stimulus-response mappings – in order to attain a higher behavioral goal. Apparently, both patient groups in our study failed to robustly activate the anterior PFC when challenged with a cognitive probe, which corroborates previous reports in MDD during a complex planning task [51] and a verbal fluency task [52]. It also concurs with frequently observed hypoactivation of DLPFC in MDD [53] and in OCD [54], which may underlie psychomotor retardation [55] as well as executive impairments in these disorders [56]. Notably, we previously also reported decreased recruitment of the anterior PFC in OCD and MDD during reversal learning [57] [32], which implies that MDD and OCD are commonly characterized by reduced recruitment of the anterior PFC during switching, either within an affective [57] [32] or cognitive context.

In the present study, the MDD group also showed reduced task-related activations in the inferior parietal cortex, corroborating a recent neuroimaging task switching study in depressed adolescents [48]. Inferior parietal involvement in task switching has been associated with attention shifting [40] and with facilitation of stimulus-response reversals during task switching (Barber & Carter, 2005). Moreover, MDD subjects showed reduced recruitment of the precuneus compared with OCD patients. Precuneus activations during task switching have been proposed to reflect attentional demands when updating stimulus-response associations [39]. Taken together, these results indicate that MDD is characterized by blunted responsiveness in attention-related brain regions compared with both controls and OCD patients. This blunted signal in attention-related brain areas during switching in MDD may underlie the previously outlined increased switch-related response latencies at a behavioral level in this group, and putatively reflect deficits in attention control at the clinical level of this disorder [58].

In contrast to brain areas that we found underactivated in the patient groups, we observed increased putamen activity in OCD compared with controls (left-lateralized) and depressed patients (bilaterally). In addition, putamen activity was correlated with OC severity as measured using Padua-IR and Y-BOCS in the OCD sample. The putamen has increasingly been associated with cognitive functions including cognitive flexibility [59] [60]. OCD has been associated with increased metabolism and regional cerebral blood flow (rCBF) in the putamen at rest [61], and with increased putamen grey matter volume [62]. A recent narrative review postulated a dysfunctional ‘compulsive’ frontal-striatal circuit in OCD, in which overactivity of the putamen (and caudate) may drive compulsive behaviors as seen in OCD [11], possibly explaining current and previous observations of putamen hyperactivity in these patients. However, we cannot rule out the alternative explanation, i.e. that switch-related hyperactivity in the putamen as found in the present study may be driven by decreased responsiveness of this structure to REs. The putamen (as part of the dorsolateral striatum) has long been associated with the forming of habits [60] (i.e. well-established stimulus-response associations), and attenuated signal in this brain region during task repetition may therefore reflect a decreased ability to implicitly learn stimulus-response associations in OCD, relative to the other groups.

As expected, we found increased task-related engagement of bilateral dorsal ACC in OCD compared with controls, and (right) dorsal ACC activity in OCD was positively correlated with Y-BOCS scores. It is well established that the ACC plays a pivotal role in ‘error monitoring’ [63] – presumably a relevant cognitive demand during task switching [14] –, as well as in mediating negative emotional states [64]. The ACC forms part of a paralimbic circuit encompassing, among other areas, the anterior insula [65]. Also as hypothesized, patients with OCD showed differential, i.e. increased anterior insula activity compared with depressed patients. The insula is important in the identification of aversive stimuli [66], and recent reviews posit the joint activation of ACC and anterior insula as the anatomical substrate of (negative) emotional awareness together with arousal-driven behavior [67] [64]. Thus, whereas reduced anterior PFC activity conjoint with increased putamen recruitment may represent the neural substrate of a switching strategy specific to OCD, we also observed increased involvement of an arousal-related paralimbic brain circuit that may reflect increased error monitoring or the ‘something is wrong’- feeling characteristic of patients with OCD [4]. The observed hyperactivity in a paralimbic circuit during events that elicit response conflict (i.e. switching) is in line with other fMRI studies in OCD that also found ACC hyperactivity in paradigms encompassing various high-conflict situations [56] [68]. In addition, a recent study in OCD likewise reported increased anterior insula activity during decision making [69], whereas insula activity was found to be decreased in adolescents with MDD during task switching [48]. The present study extends these previous results by showing that this increased paralimbic activity during high-conflict situations is unique for OCD relative to MDD. However, our finding of OCD-specific increased BOLD responses in ACC during switching is at odds with two previous neuroimaging reports on task-switching in OCD that reported hypoactivity in this brain area for these patients [46] [49]. As noted earlier, this discrepancy may be due to the use of different task switching designs, but also to different patient characteristics (i.e., medication use and gender ratio). In addition, higher levels of co-morbid depression symptoms were reported in these previous studies (mean BDI = 15.5) [49] and 18.7 [46]) compared with ours (mean BDI = 10.7). The latter may especially be relevant, since in the present study our sample of depressed patients (mean BDI = 24.9) failed to activate the ACC both in group and group x task analyses, a finding that is congruent with another recent neuroimaging task switching study in depressed adolescents [48].

The current study is not without limitations. First, we chose to implement a rapid event-related design without null events or baseline epochs similar to Sohn et al. [40], to avoid the occurrence of additional baseline to task switches, which however precluded the assessment of BOLD main effects of non-switch events. Second, groups were not adequately matched on gender, and although total Padua-IR scores were considerably higher in the OCD compared with the MDD group, between-patient group differences were not significant. However, analyses of Padua-IR subdimensions in both groups showed that MDD patients (in contrast to OCD patients) predominantly scored on rumination, a cognitive phenomenon that is itself highly characteristic of depression [58]. Moreover, the Padua-IR is known to poorly differentiate between OCD and MDD [70]. Third, although the OCD group was free of currently co-morbid depression, patients with OCD scored significantly higher than controls on depression severity measures. However, ratings on these measures in the OCD group were well below computation-based cutoff scores for clinical remission in MDD (e.g.<10 for the MADRS [71]), and a large, significant gap still remained between OCD and MDD patients on all depression scores in this study. Finally, although we used inclusive masking to reduce the risk of Type I error when performing group x condition interaction analyses, correlation analyses in MDD an OCD subjects should be considered exploratory and are therefore clearly in need of replication.

In conclusion, the present fMRI study is the first to report common and distinct behavioral and neural patterns on a ‘pure’ cognitive neuroimaging activation paradigm in OCD and MDD. In the current experiment, we used a promising neuropsychological tool, i.e. cognitive flexibility, for probing neurobiological distinctions between these disorders. Our results contribute to the process of identifying endophenotypes in complex and frequently co-morbid neuropsychiatric disorders, such as OCD and MDD. Presumably, this will lead to a better diagnostic characterization and to more specific treatments for OCD and MDD in the future.
